# Associations Between Smartphone Keystroke Metadata and Mental Health Symptoms in Adolescents: Findings From the Future Proofing Study

**DOI:** 10.2196/44986

**Published:** 2023-05-15

**Authors:** Taylor A Braund, Bridianne O’Dea, Debopriyo Bal, Kate Maston, Mark Larsen, Aliza Werner-Seidler, Gabriel Tillman, Helen Christensen

**Affiliations:** 1 Faculty of Medicine and Health University of New South Wales Kensington Australia; 2 Black Dog Institute University of New South Wales Randwick Australia; 3 Institute of Health and Wellbeing Federation University Ballarat Australia

**Keywords:** adolescents, anxiety, depression, digital phenotype, keystroke dynamics, keystroke metadata, smartphone, students

## Abstract

**Background:**

Mental disorders are prevalent during adolescence. Among the digital phenotypes currently being developed to monitor mental health symptoms, typing behavior is one promising candidate. However, few studies have directly assessed associations between typing behavior and mental health symptom severity, and whether these relationships differs between genders.

**Objective:**

In a cross-sectional analysis of a large cohort, we tested whether various features of typing behavior derived from keystroke metadata were associated with mental health symptoms and whether these relationships differed between genders.

**Methods:**

A total of 934 adolescents from the Future Proofing study undertook 2 typing tasks on their smartphones through the Future Proofing app. Common keystroke timing and frequency features were extracted across tasks. Mental health symptoms were assessed using the Patient Health Questionnaire-Adolescent version, the Children’s Anxiety Scale-Short Form, the Distress Questionnaire 5, and the Insomnia Severity Index. Bivariate correlations were used to test whether keystroke features were associated with mental health symptoms. The false discovery rates of *P* values were adjusted to *q* values. Machine learning models were trained and tested using independent samples (ie, 80% train 20% test) to identify whether keystroke features could be combined to predict mental health symptoms.

**Results:**

Keystroke timing features showed a weak negative association with mental health symptoms across participants. When split by gender, females showed weak negative relationships between keystroke timing features and mental health symptoms, and weak positive relationships between keystroke frequency features and mental health symptoms. The opposite relationships were found for males (except for dwell). Machine learning models using keystroke features alone did not predict mental health symptoms.

**Conclusions:**

Increased mental health symptoms are weakly associated with faster typing, with important gender differences. Keystroke metadata should be collected longitudinally and combined with other digital phenotypes to enhance their clinical relevance.

**Trial Registration:**

Australian and New Zealand Clinical Trial Registry, ACTRN12619000855123; https://www.anzctr.org.au/Trial/Registration/TrialReview.aspx?id=377664&isReview=true

## Introduction

Mental disorders are prevalent among adolescents worldwide and have significantly increased over recent years [1,2]. Approximately half of the mental disorders emerge before the age of 18 years [3], with more than 1 in 8 young people (ie, 4-17 years) meeting criteria for a diagnosis of a mental disorder in the last 12 months [1]. Despite the existence of evidence-based treatments, few adolescents who seek professional help receive effective treatment [4]. Of those who seek professional help, up to 50% do not respond to treatment [5]. To address the rising rates of youth mental illness, digital phenotyping (ie, using smartphone data to build a rich, personalized digital picture of behavior) has been proposed as a novel solution to enhance all aspects of clinical care, including diagnosis, symptom monitoring, and predicting response to treatment [6-8].

Among the digital phenotypes currently being developed, typing behavior, such as keystroke dynamics presents, is one of the most relevant. Keystroke dynamics refers to the detailed timing information captured when pressing and releasing of keys on a keyboard [9]. Although keystroke dynamics have historically been used as a behavioral biometric to verify individuals’ identities for security purposes (eg, phone banking and security systems), they have more recently been leveraged from individuals’ smartphones to remotely diagnose mental disorders and monitor mental health symptoms (for a review, see Alfalahi et al [10]). Typing behavior is especially relevant to adolescents, given smartphones are now almost universally owned and where typing behavior, such as instant messaging is among the most frequently used features [11-13].

Recent studies applying keystroke dynamics to mental health diagnostics and symptom monitoring have shown promising findings. For example, Cao et al [14] and Huang et al [15] first applied deep learning models to keystroke metadata to predict depression and mania symptoms in participants with bipolar disorder from the BiAffect study, yielding high predictive accuracies (~90%). Using the same sample, Stange et al [16] showed that an increased instability of typing speed (ie, root-mean-square successive difference) predicted higher future depression but not mania symptoms, whereas Zulueta, Zulueta et al [17] found that increased average interkey delay (ie, the average time between keystrokes) predicted higher depression but not mania symptoms. Mastoras et al [18] found a random forest classifier was the best-performing model among common machine learning models using various keystroke features to predict depression status. Vesel et al [19] used a larger mixed clinical sample to show higher depression was associated with more variable typing speed, and Bennett et al [20] found a random forest classifier best predicted change in depression severity (ie, change of 4 or more on the Patient Health Questionnaire [PHQ]) using keystroke features with high accuracy (ie, 90%). Together, these studies suggest that some keystroke dynamics features can provide useful information for predicting symptoms of depression and mania and diagnosing depression status.

However, these studies have several limitations. First, most of the studies have used small sample sizes (ie, n≤25 [14-18]). Second, extraction and application of keystroke timing and frequency features vary greatly between studies. Third, samples have mostly been limited to adults with bipolar disorder and outcome measures of depression and mania symptoms. Finally, all studies (except for Mastoras et al [18]) have used keystroke metadata collected during participants' routine use of their smartphone using the same platform (ie, BiAffect [17]), limiting the generalizability to other data collection methodologies (eg, task-derived metadata) and other platforms. Together, these limitations impact the validity, replicability, and translatability of the findings.

Recently, the Future Proofing study [21,22] has collected digital data from n>6000 adolescents in a high school setting to facilitate prevention and early intervention of depression. Initial findings suggest that the sample is broadly representative of the Australian adolescent population, with digital data actively and passively collected continuously throughout the study [22]. Although not all participants were involved in typing data collection, data from this study provides an excellent opportunity to explore keystroke features associated with mental health symptom severity in a large sample using various keystroke features across a range of mental health measures.

This paper aims to examine the associations between common keystroke timing and frequency features and mental health symptoms in adolescence using data from the Future Proofing study [21,22]. Keystroke typing features were derived from 2 typing tasks completed by 934 participants in the custom-built Future Proofing app within 2 weeks of their baseline assessment. It was hypothesized that increased severity in depression symptoms would be associated with slower keystrokes and less frequent keystrokes due to increased psychomotor impairment [17,18]. It was also hypothesized that machine learning models using keystroke features would significantly predict symptoms of depression [14,15,18,20]. Relationships between keystroke features and other mental health symptoms (ie, anxiety, distress, and insomnia) were exploratory.

## Methods

### Design

This study was a secondary analysis of baseline data from a prospective cohort study with an embedded cluster randomized controlled trial. The trial protocol [21] and baseline characteristics [22] have been published elsewhere. Ethics approvals were obtained from the University of New South Wales Human Research Ethics Committee (HC180836), the State Education Research Applications Process for the New South Wales Department of Education (SERAP2019201), and relevant Catholic Schools Dioceses across Australia.

### Setting

The Future Proofing study was conducted in 134 secondary schools located across Australia, including government and nongovernment (independent and Catholic) schools. Recruitment was conducted from March 2019 to March 2022. All New South Wales government and independent secondary schools and eligible New South Wales Catholic secondary schools were invited to participate. Independent schools in capital cities from around Australia were also invited to participate. Consent was provided on the internet from a parent or guardian and the adolescent before study participation. Data collection took place across 3 separate year 8 cohorts (students aged 13-14 years): the 2019 Cohort from August to September 2019; the 2020 Cohort from August to November 2020; the 2021 Cohort from April-December 2021 (extended to March 2022 due to COVID-19).

### Participants

Participants (N=6388) were secondary school adolescents from year 8 who attended participating schools. All adolescents enrolled in year 8 at each participating school were eligible to participate in the trial if they had a smartphone with iOS or Android operating system and an active phone number. All participants were invited to undertake the typing tasks, as part of an omnibus of digital tasks [22]. Participants were limited to those who completed the typing speed tasks and had their digital data available for analysis (1208/6388, 18.9%) and those who completed the tasks within the first 2 weeks of completing their baseline questionnaires (ie, 934/1208, 77.3%; for distributions of days between baseline assessment and typing task completion, see Figure S1 in Multimedia Appendix 1).

### Procedure

Participants with parental consent completed the study questionnaires through a secure web-based portal, accessible using their phone number and a 1-time password sent through SMS text messaging. Following the completion of baseline questionnaires, participants were instructed to open the Future Proofing app and complete the typing speed tasks during class time and in their own time.

### Typing Task

Participants completed 2 typing tasks through the Future Proofing app—the prose task and the composition task. During the prose task, participants were asked to copy (type) as much text from a given script as possible within 30 seconds. For the composition task, participants were asked to type between 200 and 250 characters on a randomly given topic with no time constraints. Scripts and topics were randomly allocated to participants from a pool of 8 (see Figure S2 and Text S1 in Multimedia Appendix 1 for task screenshots, scripts, and topics).

### Measures

#### Depressive Symptoms

The PHQ for Adolescents (PHQ-A) is a validated modification of the PHQ-9 for adolescents, a 9-item self-administered depression severity screening and diagnostic tool based on Diagnostic and Statistical Manual of Mental Disorders, fourth edition (DSM-IV) criteria [23]. The scale assesses the frequency of occurrence of 9 depression symptom criteria during the previous 2 weeks, with items rated on a 4-point scale ranging from 0 “Not at all” to 3 “Nearly every day.” Total scale scores on the PHQ-A depression scale can range from 0 to 27, with higher scores reflecting more severe depressive symptoms. The accepted clinical cutoff points are as follows: a score of 0-4 indicates nil to minimal symptoms, 5-9 indicates mild symptoms, 10-14 indicates moderate symptoms, 15-19 indicates moderately severe symptoms and 20-27 indicates severe symptoms. A threshold of ≥15, reflecting moderately severe symptoms was used to determine caseness. The internal consistency of the PHQ-A in this study was high (*α*=.88 [22]).

#### Anxiety Symptoms

The Spence Children’s Anxiety Scale Short-Form (CAS-8) is an 8-item brief measure of anxiety for children and adolescents, based on the Spence Children’s Anxiety Scale [24]. The Spence Children’s Anxiety Scale was designed to measure the severity of children's and adolescents’ anxiety symptoms based broadly on Diagnostic and Statistical Manual of Mental Disorders, fourth edition criteria for anxiety disorders [25]. Respondents rate the degree to which they experience each symptom on a 4-point frequency scale, ranging from 0 “Never” to 3 “Always.” Total scale scores on the 8-item CAS-8 can range from 0 to 24, with higher scores reflecting greater anxiety. A threshold of ≥14 was used to determine caseness. The internal consistency of the CAS-8 in this study was high (*α*=.88 [22]).

#### Distress

The Distress Questionnaire-5 (DQ-5; [26]) is a 5-item brief screening tool for identifying general psychological distress. Respondents rate each item on a 5-point scale, ranging from 1 “Never” to 5 “Always.” The total scores on the scale range from 5 to 25, with higher scores indicating greater psychological distress. A threshold of ≥14 was used to determine high distress caseness. The internal consistency of the DQ-5 in this study was high (*α*=.88 [22]).

#### Insomnia

The Insomnia Severity Index (ISI) is a psychometrically sound, 7-item self-report measure of insomnia symptoms over the previous 2 weeks [27]. Responses are reported on a Likert scale ranging from 0 “Not at all” to 4 “Very,” producing total scores of 0-28. Cutoff scores are as follows: 0-7 reflects no clinically significant insomnia, 8-14 indicates subthreshold insomnia, 15-21 suggests moderate severity insomnia, and 22-28 indicates severe insomnia. A threshold of ≥15, reflecting moderately severe symptoms was used to determine high insomnia caseness. The ISI was designed for use in adults but has been widely administered to, and validated in, adolescent samples [28,29].

### Preprocessing and Feature Extraction

Data were restricted to participants who completed their first composition and prose tasks within 2 weeks from their baseline questionnaires. In total, 54 features were extracted (for a full list, see Table S1 in Multimedia Appendix 1). For the keystroke timing features of dwell (ie, the time interval between a key press and release of the same key), latency (ie, the time interval between a key press of a keystroke and key release of the following keystroke), interval (ie, the time interval between a key press of a keystroke and key release of the following key), up up (ie, the time interval between the key release of a keystroke and key release of the following keystroke), and down down (ie, the time interval between key press of a keystroke and key press of the following keystroke), extracted features included the median, mean, variance, minimum, maximum, Q1, Q3, and skewness, kurtosis. Keystroke frequency features include total keystrokes, total backspace, total spaces, total nonalphanumeric, and total null keys (ie, unlabeled or undetermined keys), as well as the proportion of total backspace, total spaces, total nonalphanumeric, and total null keys to total keystrokes. Features were extracted using metadata from both typing tasks, as well as separately from the task to investigate whether the task impacted the relationship between keystroke features and mental health symptoms.

### Statistical Analysis

Pearson correlations were used to test associations between keystroke features and mental health symptoms. The false discovery rate of *P* values was adjusted to *q* values using the Benjamini and Hochberg [30] approach. Correlations were also tested separately by gender (restricted to males and females due to limited numbers in other categories) and likely clinical caseness to investigate whether these groups differed in their patterns of association.

Various machine learning models including linear and nonlinear support vector machines, neural nets, random forest, extreme gradient boosting, and elastic net models were used to test whether all keystroke features and demographic variables could be combined to predict mental health symptoms. Machine learning models were chosen based on their performance and use among the keystroke dynamics literature [18,20,31] and more widely in precision psychiatry [32,33]. Highly correlated features (*r*>0.95) were first removed from the 54 keystroke features, leaving 34 features. Data were then split into training (80%) and test (20%) sets so models developed using the training set could be validated through hold-out cross-validation in the test set. Hyperparameters were manually optimized using logarithmically spaced values between 2^–10^ and 2^10^ where appropriate (except for random forest models, where the number of features was set between 1:34) in the training set using 10-fold cross-validation with 20 repeats based on the root mean standard error with and without recursive feature elimination. The best-performing models were then validated on the test set. Model performance was assessed by correlating the predicted values with the observed values in the test sets. Models were also developed with the demographic variables gender and age included among the pool of features to determine whether demographic variables could improve model performance beyond keystroke features alone.

All analyses were performed using R 4.2.1 [34]. Correlation plots were created using the “corrplot” package in R [35]. Machine learning models were trained using the “caret” package in R [36].

## Results

### Participants

Participant characteristics are presented in Table 1. The mean age was 13.8 (SD 0.6, range 11.1-16.3) years and 64.0% (598/934) were female. The mean PHQ-A score was 7.9 (SD 6.5), with 16.8% (157/934) meeting the clinical cut-off for depression (for distributions of mental health scores, see Figure S3 in Multimedia Appendix 1). Latency had the longest duration (mean 0.30, SD 0.08) and dwell had the shortest duration (mean 0.08, SD 0.01) among keystroke timing features. The mean total keystrokes was 98.5 (SD 37.1) with few nonalphanumeric (mean 1.7, SD 1.4) or null keys (mean 0.4, SD 1.3) used. Most participants self-reported that they typed with both hands (72.7%; 679/934) and 34.4% (321/934) used iPhones.

**Table 1:** Demographic and clinical characteristics of participants.

Characteristics			Total sample (N=934)
Age (years), mean (SD)			13.84 (0.56)
Depressive symptoms (PHQ-A^a^), mean (SD)			7.87 (6.5)
Anxiety symptoms (CAS-8^b^), mean (SD)			9.11 (5.6)
Distress symptoms, mean (SD)			11.65 (5.1)
Insomnia symptoms, mean (SD)			7.62 (5.86)
**Keystroke timing features (median), mean (SD)**			
	Dwell		0.08 (0.01)
	Latency		0.30 (0.07)
	Interval		0.14 (0.06)
	Up up		0.21 (0.07)
	Down down		0.22 (0.07)
**Keystroke frequency features (total), mean (SD)**			
	Keystrokes		337.97 (168.54)
	Spaces		57.15 (31.93)
	Backspaces		42.01 (36.75)
		Nonalphanumeric	4.66 (7.39)
		Null keys	0.92 (3.55)
Gender (female), n (%)			598 (64)
**Typing hand, n (%)**			
	Left		8 (0.9)
	Right		60 (6.4)
	Both		679 (72.7)
	Not reported		187 (20)
**Device, n (%)**			
	iOS		321 (34.4)
	Android		98 (10.5)
	Not reported		515 (55.1)
**Likely clinical case, n (%)**			
	Depressive symptoms (PHQ-A) (≥15)		157 (16.8)
	Anxiety symptoms (CAS-8) (≥14)		208 (22.3)
	Distress symptoms (DQ-5^c^) (≥14)		317 (34)
	Insomnia symptoms (ISI^d^) (≥15)		133 (14.3)

^a^PHQ-A: Patient Health Questionnaire for Adolescents.

^b^CAS-8: Children’s Anxiety Scale-Short Form.

^c^DQ-5: Distress Questionnaire-5.

^d^ISI: Insomnia Severity Index.

### Associations Between Keystroke Features and Mental Health Symptoms

**Figure 1:**
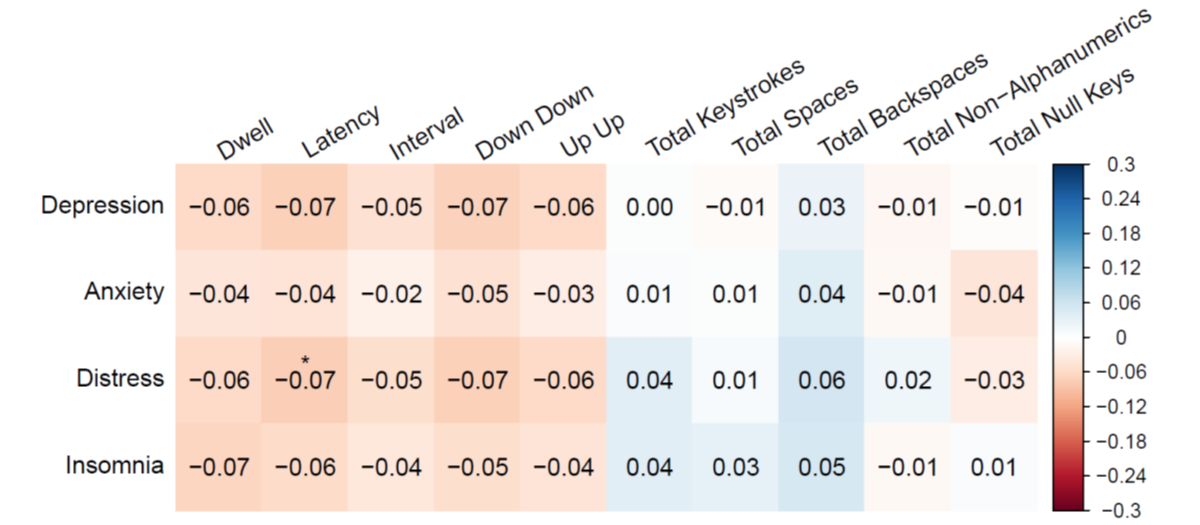
Associations between mental health symptoms and common keystroke features.

Associations between mental health symptoms and commonly reported keystroke features (ie, median timing and total frequency features) are presented in Figure 1 (for all features, see Figures S4 and S5 in Multimedia Appendix 1). Keystroke metadata was combined across tasks and prompts due to similar patterns of association being observed when assessing associations separately by task (see Figure S6 in Multimedia Appendix 1) and by emotionally valanced prompts (see Figure S7 in Multimedia Appendix 1). All measures of mental health symptoms (ie, depression, anxiety, distress, and insomnia) showed weak negative associations with all keystroke timing features, indicating that increased mental health symptoms were associated with faster typing. However, after correcting for multiple comparisons, only the association between distress and latency remained significant (*r*=0.07, *q*=0.045). Measures of mental health symptoms showed a mix of weak positive and negative associations with keystroke frequency features, although none of these relationships were significant (all *q*>0.05).

**Figure 2:**
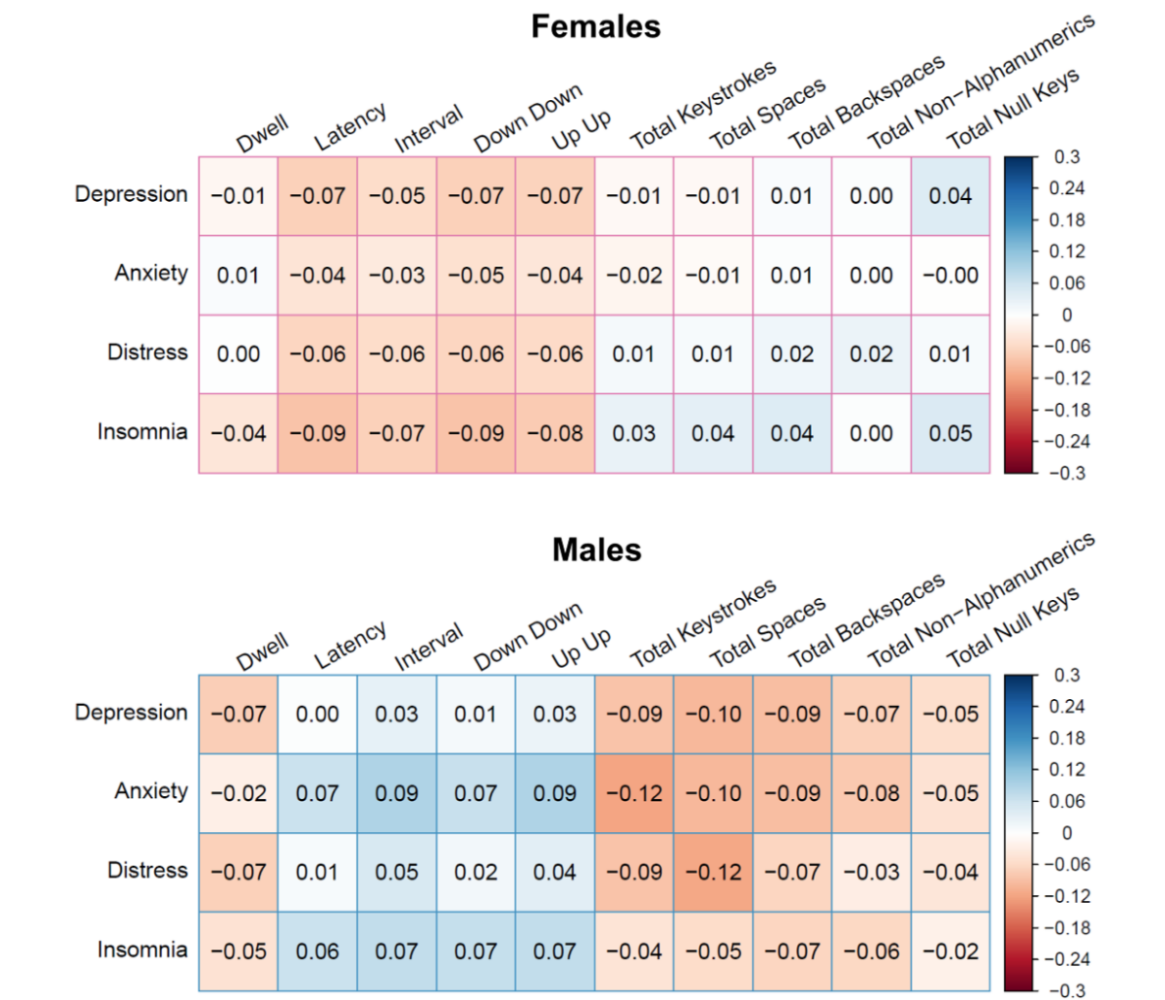
Associations between mental health symptoms and keystroke features for females and males.

Associations between mental health symptoms and keystroke features by gender are presented in Figure 2 (for all features, see Figures S8-S11 in Multimedia Appendix 1). In females, a similar pattern emerged between mental health symptoms and keystroking timing features, whereby increased mental health symptoms were associated with faster typing. Generally, positive associations were also seen between mental health symptoms and keystroke frequency features, whereby increased mental health symptoms were associated with more frequent keystrokes. In males, the opposite patterns emerged with mental health symptoms showing a weak positive association with keystroke timing features (except for dwell) and weak negative associations with timing features. However, all associations were nonsignificant after correcting for multiple comparisons (all *q*>0.05).

**Figure 3:**
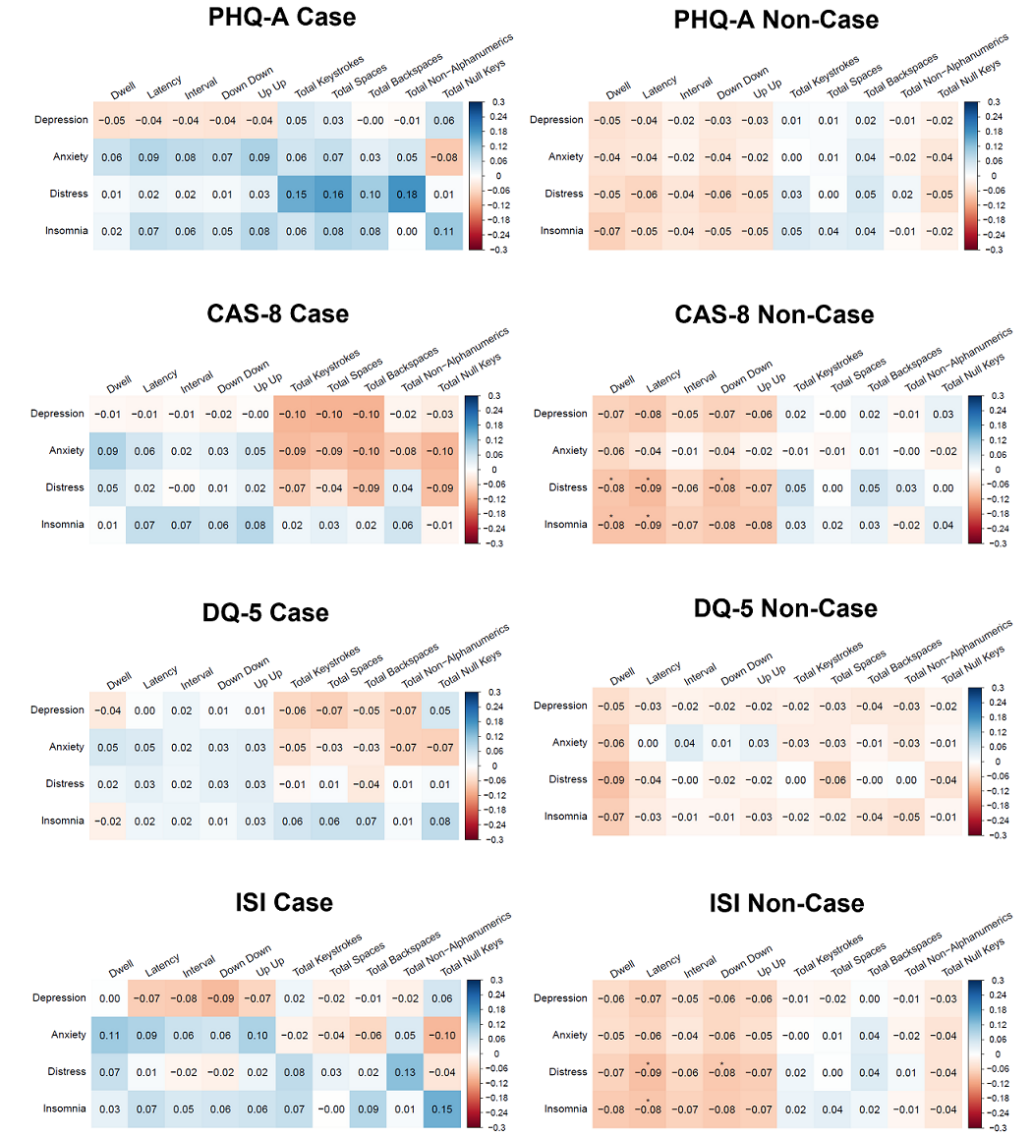
Associations between mental health symptoms and common keystroke features for people who do and do not meet likely clinical caseness. CAS-8: Children’s Anxiety Scale-Short Form; DQ-5: Distress Questionnaire-5; ISI: Insomnia Severity Index; PHQ-A: Patient Health Questionnaire for Adolescents.

Associations between mental health symptoms and keystroke features by likely clinical caseness are presented in Figure 3 (for all features, see Figures S12-S16 in Multimedia Appendix 1). In people who did not meet PHQ-A caseness, all measures of mental health symptoms showed weak negative associations with all keystroke timing features, whereas people who did meet PHQ-A caseness showed the opposite pattern (except for depression). All measures of mental health symptoms generally showed weak positive associations with all keystroke frequency features in both people who did and did not meet PHQ-A caseness (with the exception of null keys). However, all associations were nonsignificant after correcting for multiple comparisons (all *q*>0.05).

In people who did not meet CAS-8 caseness, all measures of mental health symptoms showed weak negative associations with all keystroke timing features, with significant associations between distress and dwell (*r*=−0.08, *q*=0.048), latency (*r*=−0.09, *q*=0.026), and down down (*r*=–0.08, *q*=0.045), as well as insomnia and dwell (*r*=−0.08, *q*=0.042) and latency (*r*=−0.09, *q*=0.037). People who did meet CAS-8 caseness showed the opposite pattern (except for depression). All measures of mental health symptoms generally showed weak positive associations with all keystroke frequency features in people who did not meet CAS-8 caseness, whereas the opposite pattern was seen in people who did meet CAS-8 caseness. However, these associations were nonsignificant after correcting for multiple comparisons (all *q*>0.05).

In people who did not meet DQ-5 caseness, all measures of mental health symptoms showed weak negative associations with all keystroke features. In people who did meet DQ-5 caseness, all measures of mental health symptoms showed weak positive associations with all keystroke timing features and weak negative associations between mental health symptoms (except for insomnia) with keystroke frequency features. However, all associations were nonsignificant after correcting for multiple comparisons (all *q*>0.05).

In people who did not meet criteria for ISI caseness, all measures of mental health symptoms showed weak negative associations with all keystroke timing features, with significant associations between distress and latency (*r*=−0.09, *q*=0.023) and down down (*r*=−0.08, *q*=0.046), as well as insomnia and latency (*r*=−0.08, *q*=0.035). People who did meet ISI caseness showed the opposite pattern (except for depression). Both people who did and did not meet criteria for ISI caseness showed a mix of weak positive and negative associations between mental health symptoms and keystroke frequency features. However, these associations were nonsignificant after correcting for multiple comparisons (all *q*>0.05).

### Keystroke Features Predicting Mental Health Symptoms

Results from machine learning models using keystroke features and demographics to predict mental health symptoms are presented in Table 2. The best-performing models for predicting mental health symptoms were elastic net models. No significant models could be produced using keystroke features alone (all *P*>.05). When using keystroke features and gender, predicted and observed values were correlated for depression (*r*=0.30, *P*<.001), anxiety (*r*=0.38, *P*<.001), distress (*r*=.33, *P*<.001), and insomnia (*r*=0.19, *P*=.01) in the test sets. However, keystroke features were minimally weighted compared to gender (see Figure S17 in Multimedia Appendix 1). Models were also developed using additional multiplicative features between gender and keystroke features to test whether including these additional features would improve prediction accuracy, but these models were similarly poor performing. No significant models could be produced when developed separately by gender (all *P*>.05).

**Table 2:** Results from machine learning models where predicted values significantly correlated with observed values in test sets to predict mental health symptoms.

Mental health symptoms	Model	Hyperparameters, *α*^a^, *λ*^b^	Performance metrics		Correlation between observed and predicted test values	
			RMSE^c^	MAE^d^	*r*	*P* value
Depression	Elastic Net	1, 0.50	6.22	5.10	0.30	<.001
Anxiety	Elastic Net	8, 0.25	5.23	4.25	0.38	<.001
Distress	Elastic Net	1, 0.25	4.75	3.93	0.33	<.001
Insomnia	Elastic Net	125, 0.5	5.68	4.65	0.19	.01

^a^*α*: alpha.

^b^*λ*: lambda.

^c^RMSE: root-mean-square error.

^d^MAE: mean absolute error.

### Supplemental Analysis of Total Keystrokes and Total Spaces Split by Task

Although most keystroke features showed similar patterns across tasks, we performed additional supplemental analyses of the frequency features “total keystrokes” and “total spaces” due to their stronger associations with mental health symptoms in the prose task when compared to the composition task. Although females show similar patterns of association between composition and prose tasks for total keystrokes and total spaces, males showed different patterns. However, none of the associations split by gender and task were significant (see Figure S18 in Multimedia Appendix 1). Noncases showed similar patterns of association between composition and prose tasks for total keystrokes and total spaces. However, cases showed different patterns between tasks, with a significant association between insomnia and total spaces for the composition task (*r*=0.22, *q*=0.032). No other associations split by caseness and task were significant (see Figure S19 in Multimedia Appendix 1).

## Discussion

### Overview

This study investigated associations between common keystroke timing and frequency features and mental health symptoms in a large cohort of adolescents. We found increased mental health symptoms (ie, depression, anxiety, distress, and insomnia) were weakly associated with faster keystroke timing features. When split by gender, females with higher mental health symptoms exhibited faster keystroke timing features and fewer keystroke frequency features, whereas males showed opposite patterns. When split by likely clinical caseness, various patterns of association were found. However, most associations were no longer significant following corrections to multiple comparisons. Machine learning models using keystroke features alone also did not predict mental health symptoms and contributed minimally when models included gender.

Few studies have directly assessed correlations between keystroke features and mental health symptoms [16,20]. Of those studies, only Bennett et al [20] provided a correlation matrix assessing associations between a range of keystroke features and depression severity (measured using the PHQ-8) and similarly found a mix of weak positive and negative associations (*r*=0.16 to 0.10). Our finding of increased mental health symptoms being associated with faster keystroke timing features is in line with suggestions that increased psychomotor agitation could lead to a general speeding up of behaviors, including typing speed [17]. However, increased mental health symptoms, such as depression can also lead to psychomotor impairment, which will produce slower typing speeds. The diverse symptom profiles experienced by people with mental disorders and differences in associations between various subgroups seen in this study highlight the need for more personalized approaches to assessment and treatment [6,7,37-39]. The relative weakness of cross-sectional associations between keystroke features and mental health symptoms also highlights the importance of needing to combine various digital phenotyping features in prediction models.

Previous studies leveraging keystroke features to predict mental health symptoms using machine learning and deep learning models have all yielded high predictive accuracies (~90%) [14,15,18,20]. The poor predictive performance yielded from the models in our study could be attributed to several factors. First, all previous studies (except for Bennett et al [20]) used small sample sizes (ie, n≤25 [14,15,18]). And while Bennett et al [20] used a larger sample size with methods to rebalance the data, no previous study used independent data sets to evaluate their models performance, which can strongly bias performance estimates [40]. Second, our models used cross-sectional task-derived keystroke metadata and measures of mental health symptoms, whereas previous studies have used session-level keystroke data collected during routine use to predict future mental health symptoms [14,15,18,20], which provides a richer pool of predictive features. Third, most studies dichotomized mental health symptoms rather than treating the measures as continuous (eg, Hamilton Depression Rating Scale scores ≤7 as negative depression and >7 as positive [14]), losing important information and ignoring the variation within each group [41]. Lastly, most studies leveraged data sets made up of a single clinical population [14,15,18], increasing uniformity and impacting generalizability. Together, our findings suggest that keystroke features provide minimal use in predicting mental health symptoms cross-sectionally.

### Limitations

This study includes several limitations. Keystroke metadata was collected from typing tasks completed within 2 weeks of participants’ baseline assessments and metadata collected temporally closer to their baseline assessment may provide more accurate reflections of mental health symptoms. However, most participants completed the typing task on the same day as their baseline assessments (see Figure S1 in Multimedia Appendix 1). Metadata related to the distance between keys was not collected, so additional features previously calculated (eg, speed [18]) could not be replicated. We combine metadata across typing tasks and prompts. Although patterns of association were generally similar across tasks and prompts, some differences in patterns of association were observed for total keystrokes and total spaces when split by task. Future studies should consider how these and other methodological considerations (eg, text length and typing accuracy) may impact associations between keystroke features and mental health symptoms. As the Future Proofing study is still being completed, longitudinal typing and mental health data were not yet available for analysis.

### Conclusions

In conclusion, our findings suggest keystroke features are weakly associated with mental health symptoms with differing patterns for males and females. Keystroke features also provide minimal clinical use in predicting mental health symptoms cross-sectionally. Future research should focus on collecting keystroke metadata longitudinally and combining these data with other digital phenotypes being developed to enhance their potential for aiding clinical care.

## Appendix

Multimedia Appendix 1 Supplementary text, figures, and tables.

## Abbreviations

CAS-8: eight-item Children’s Anxiety Scale
DQ-5: Distress Questionnaire-5
DSM-IV: Diagnostic and Statistical Manual of Mental Disorders, fourth edition
ISI: Insomnia Severity Index
PHQ-A: Patient Health Questionnaire for Adolescents
