# Supplementary material for: Associations Between Smartphone Keystroke Metadata and Mental Health Symptoms in Adolescents: Findings From the Future Proofing Study
Source: JMIR Ment Health. 2023 May 15;10:e44986. doi: 10.2196/44986 (PMC10227695; doi:10.2196/44986)
Supplement: Multimedia Appendix 1 [file mental_v10i1e44986_app1.docx]

**Supplementary Material**

**Figure S1.** Difference in days between baseline assesment and composition/prose typing task completion.


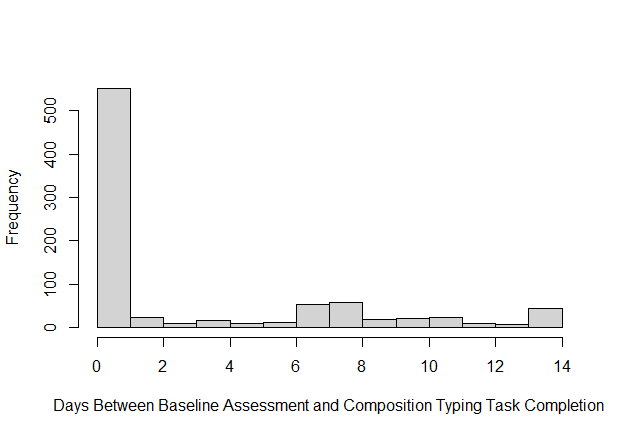

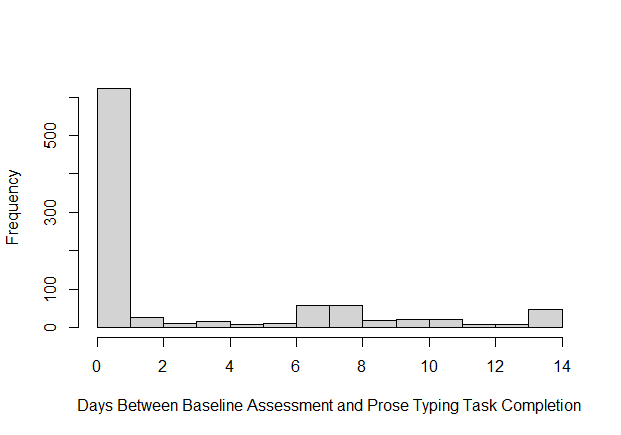


**Figure S2.** Screen shots from the prose and composition typing tasks contained in the Future Proofing Trial App.


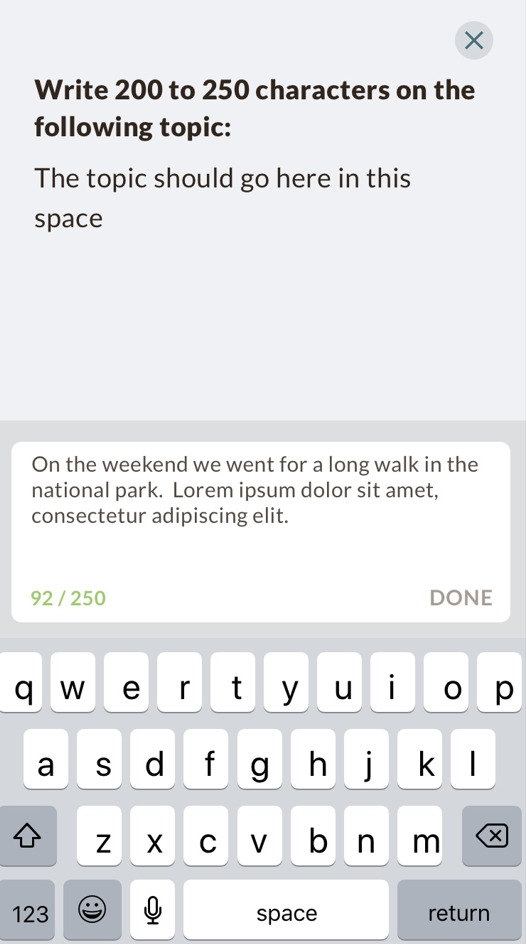

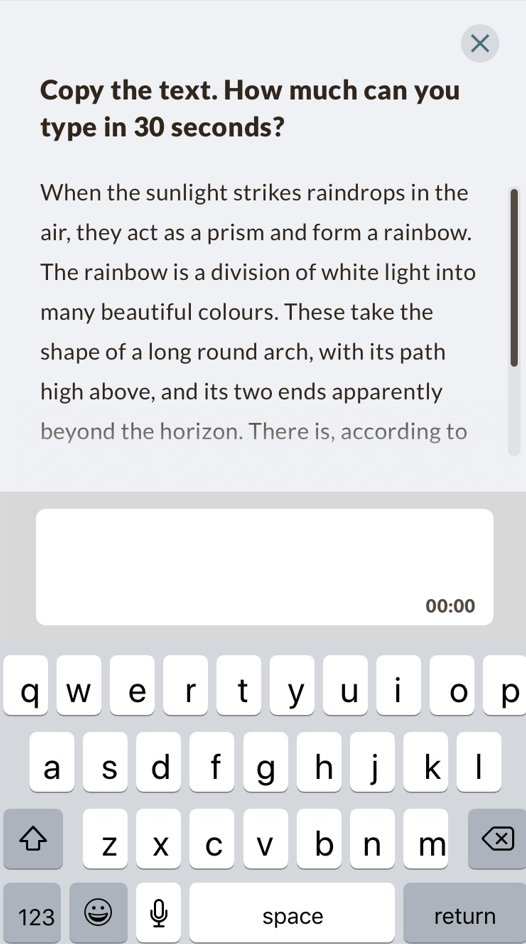

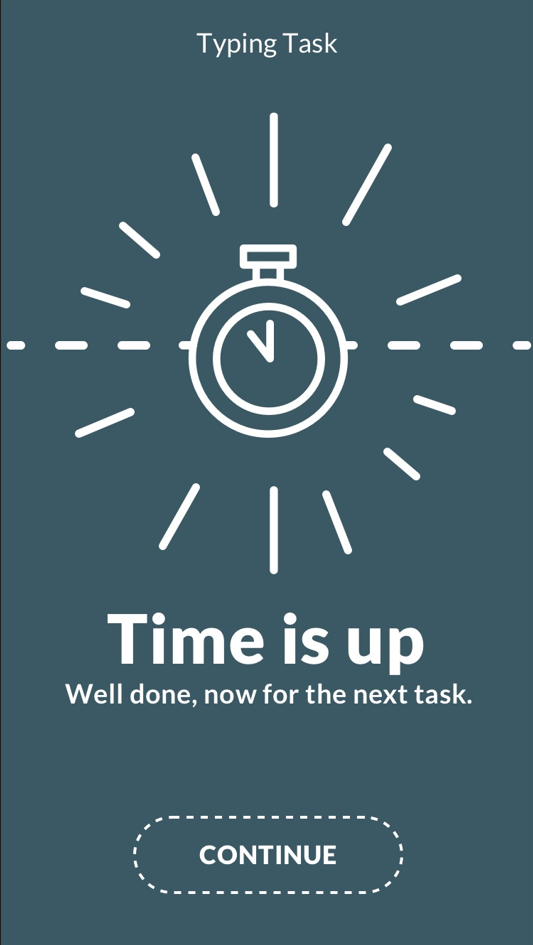

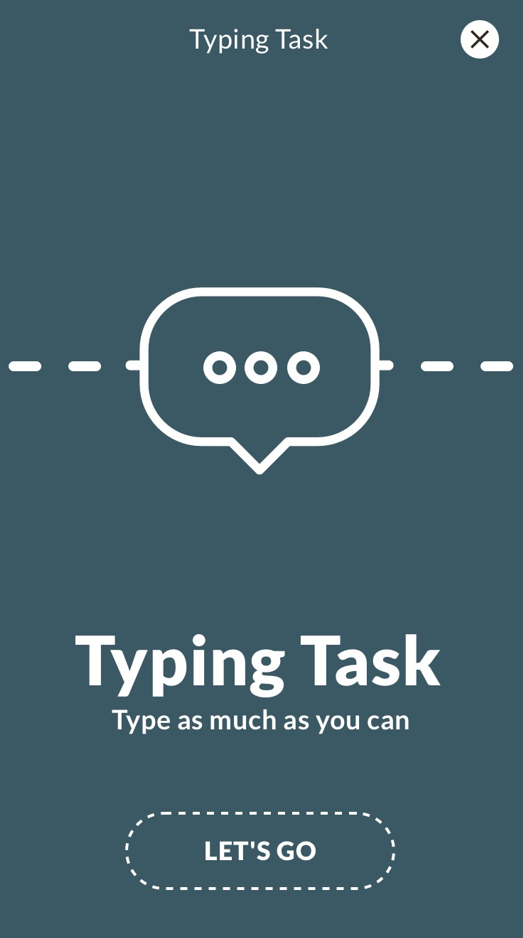


**Text S1.** Text from typing task outlining the various topics (for the composition task) and script (for the prose task).

**Topics included**: “The weather today”; “What I did yesterday”; “My favourite food”; “A time I was happy”; “A time I was sad”; “What I do in my spare time”; “My hero”; “My hopes for the future”.

**Scripts included**; “For a minute or two she stood looking at the house, and wondering what to do next, when suddenly a footman in livery came running out of the wood and rapped loudly at the door with his knuckles. She considered him to be a footman because he was in livery. Otherwise, judging by his face only, she would have called him a fish. It was opened by another footman in livery, with a round face, and large eyes like a frog; and both footmen, Alice noticed, had powdered hair that curled all over their heads. She felt very curious to know what it was all about and crept a little way out of the wood to listen. The Fish-Footman began by producing from under his arm a great letter, nearly as large as himself, and this he handed over to the other.”

“There was a table set out under a tree in front of the house, and the March Hare and the Hatter were having tea at it. A Dormouse was sitting between them, fast asleep, and the other two were using it as a cushion, resting their elbows on it, and talking over its head. The table was a large one, but the three were all crowded together at one corner of it. Alice sat down in a large armchair at one end of the table. Alice looked all round the table, but there was nothing on it but tea. The party sat silent for a minute, while Alice thought over all she could remember about ravens and writing-desks, which wasn\'t much. The Hatter was the first to break the silence. He had taken his watch out of his pocket, and was looking at it uneasily, shaking it every now and then, and holding it to his ear.”

“So much I\'ll tell you, and no more. I were in Flint\'s ship when he buried the treasure, he and six along. Six strong seamen. They was ashore nigh on a week, and us standing off and on in the old Walrus. One fine day up went the signal, and here come Flint by himself in a little boat, and his head done up in a blue scarf. The sun was getting up, and mortal white he looked about the cutwater. But, there he was, you mind, and the six all dead. Dead and buried. How he done it, not a man aboard us could make out. It was battle, murder, and sudden death, leastways. Him against six. Billy Bones was the mate, Long John, he was quartermaster, and they asked him where the treasure was.”

“Then the sound of shouting arose again and the crackle of dried thorns. The enemy was breaking down the hedge. All the villagers swarmed to the point whence the crackling and the shouting came. They hurled stones over the hedges, and short arrows with flint heads. The children had never before seen men with the fighting light in their eyes. It was very strange and terrible and gave you a thick feeling in your throat. It was quite different from the pictures of fights in the illustrated papers at home. It seemed that the shower of stones had driven back the besiegers. The besieged drew breath, but at that moment the shouting and the crackling arose on the opposite side of the village and the crowd hastened to defend that point, and so the fight swayed to and fro across the village, for the besieged had not the sense to divide their forces as their enemies had done.”

“They were friends in a friendship closer than brotherhood. Nello was a little Ardennois. Patrasche was a big Fleming. They were both of the same age by length of years, yet one was still young, and the other was already old. They had dwelt together almost all their days. Both were orphaned and destitute, and owed their lives to the same hand. It had been the beginning of the tie between them, their first bond of sympathy; and it had strengthened day by day, and had grown with their growth, firm and indissoluble, until they loved one another very greatly. Their home was a little hut on the edge of a little village—a Flemish village a league from Antwerp, set amidst flat breadths of pasture and corn-lands, with long lines of poplars and of alders bending in the breeze on the edge of the great canal which ran through it.”

“The winter was very sharp already. That night, after they reached the hut, snow fell, and fell for very many days after that, so that the paths and the divisions in the fields were all obliterated, and all the smaller streams were frozen over, and the cold was intense upon the plains. Then, indeed, it became hard work to go round for the milk while the world was all dark, and carry it through the darkness to the silent town. Hard work, especially for Patrasche, for the passage of the years, that were only bringing Nello a stronger youth, were bringing him old age, and his joints were stiff and his bones ached often. But he would never give up his share of the labour. Nello would fain have spared him and drawn the cart himself, but Patrasche would not allow it.”

“A little girl, radiant and beautiful, shapely as a fairy and exquisitely dressed, was dancing gracefully in the middle of the lonely road, whirling slowly this way and that, her dainty feet twinkling in sprightly fashion. She was clad in flowing, fluffy robes of soft material that reminded Dorothy of woven cobwebs, only it was coloured in soft tintings of violet, rose, topaz, olive, azure, and white, mingled together most harmoniously in stripes which melted one into the other with soft blendings. Her hair was like spun gold and flowed around her in a cloud, no strand being fastened or confined by either pin or ornament or ribbon. Filled with wonder and admiration our friends approached and stood watching this fascinating dance. The girl was no taller than Dorothy, although more slender, nor did she seem any older than our little heroine.”

“But they by no means suddenly stopped. Little Walter Ashe\'s case proved to be rather a severe one, and after he had begun to mend, he caught cold somehow and was taken worse again. There were some serious symptoms, and for a few days Dr. Carr did not feel sure how things would turn. He did not speak of his anxiety at home, but kept silence and a cheerful face, as doctors know how to do. Only Katy, who was more intimate with her father than the rest, guessed that things were going gravely at the other house, and she was too well trained to ask questions. The threatening symptoms passed off, however, and little Walter slowly got better. But it was a long convalescence, and Mrs. Ashe grew thin and pale before he began to look rosy.”

**Figure S3.**


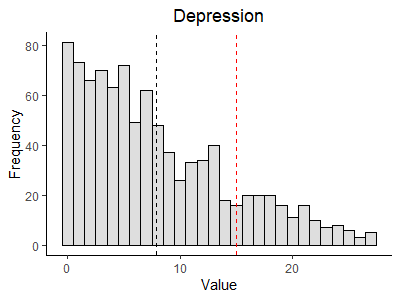

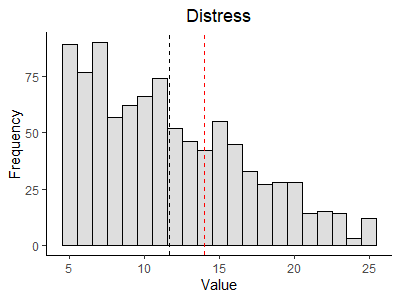

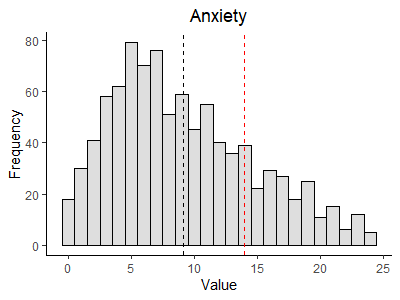

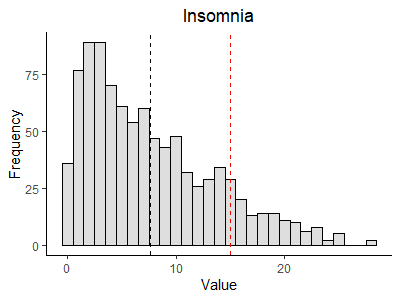


Note: Mean scores are marked by black dashed lines and clinical cut offs scores are marked by red dashed lines.

**Figure S4.** Associations between mental health symptoms and other keystroke timing features (i.e., mean, sd, min, max, q1, q3, skew, and kertosis).

6

**Figure S5.** Associations between mental health symptoms and other keystroke frequency features (i.e., frequency propotions).

**Figure S6.** Associations between mental health symptoms and keystroke features split by task.

**Prose**

**Composition**

**Figure S7.** Associations between mental health symptoms and keystroke features split by removal of emotionally valenced (i.e., “I time I was sad” and “a time I was happy”) prompts.

**All Prompts**

**No Emotionally Valanced Prompts**

**Figure S8.** Associations between mental health symptoms and other keystroke timing features for females (i.e., mean, sd, min, max, q1, q3, skew, and kertosis).

**Figure S9.** Associations between mental health symptoms and other keystroke frequency features for females (i.e., frequency propotions).

**Figure S10.** Associations between mental health symptoms and other keystroke timing features for males (i.e., mean, sd, min, max, q1, q3, skew, and kertosis).

**Figure S11.** Associations between mental health symptoms and other keystroke frequency features for males (i.e., frequency propotions).

**Figure S12.** Associations between mental health symptoms and other keystroke timing features by likely clinical caseness (i.e., mean and sd).

**PHQ-9 Case PHQ-9 Non-Case**

**CAS-8 Case CAS-8 Non-Case**

**DQ-5 Case DQ-5 Non-Case**

**ISI Case ISI Non-Case**

**Figure S13.**

Associations between mental health symptoms and other keystroke timing features by likely clinical caseness (i.e., min and max).

**PHQ-9 Case PHQ-9 Non-Case**

**CAS-8 Case CAS-8 Non-Case**

**DQ-5 Case DQ-5 Non-Case**

**ISI Case ISI Non-Case**

**Figure S14.**

Associations between mental health symptoms and other keystroke timing features by likely clinical caseness (i.e., q1 and q3).

**PHQ-9 Case PHQ-9 Non-Case**

**CAS-8 Case CAS-8 Non-Case**

**DQ-5 Case DQ-5 Non-Case**

**ISI Case ISI Non-Case**

**Figure S15.**

Associations between mental health symptoms and other keystroke timing features by likely clinical caseness (i.e., skew, and kertosis).

**PHQ-9 Case PHQ-9 Non-Case**

**CAS-8 Case CAS-8 Non-Case**

**DQ-5 Case DQ-5 Non-Case**

**ISI Case ISI Non-Case**

**Figure S16.**

Associations between mental health symptoms and other keystroke frequency features by likely clinical caseness (i.e., frequency propotions).

**PHQ-9 Case PHQ-9 Non-Case**

**CAS-8 Case CAS-8 Non-Case**

**DQ-5 Case DQ-5 Non-Case**

**ISI Case ISI Non-Case**

**Figure S17.**

Variable importance (i.e., coefficients) for models using keystroke features and gender to predict mental health symptoms.

**Depression Anxiety**

**
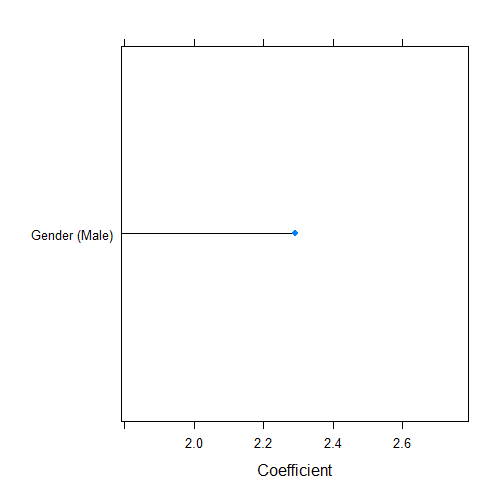

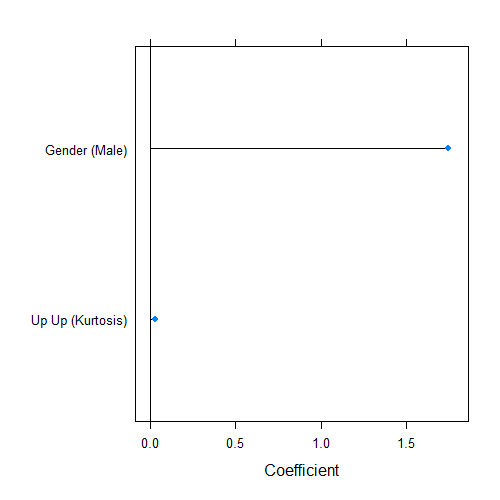
**

**Distress Insomnia**


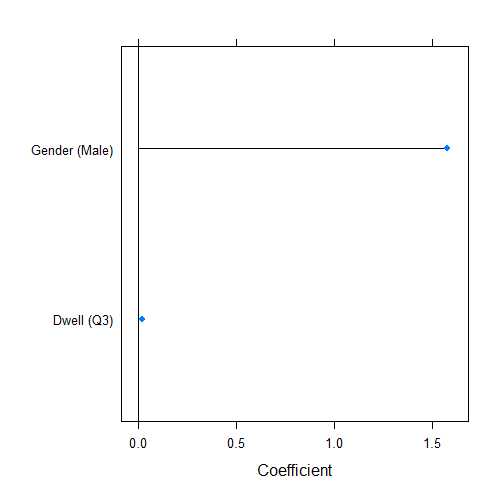
 **
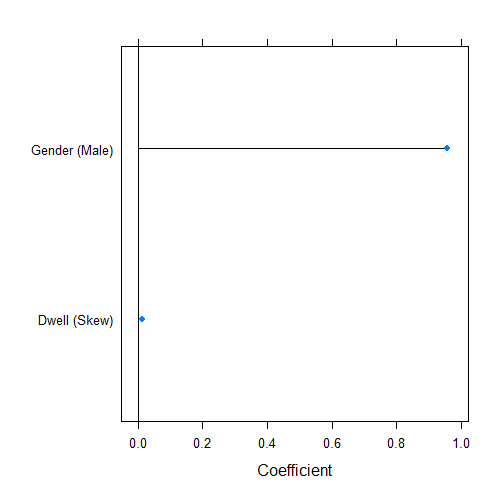
**

**Figure S18.**

Association between total keystrokes/total spaces and mental health symptoms when split by gender and task.

**Female**

**Prose Composition**

**Males**

**Prose Composition**

**Figure S19.**

Association between total keystrokes/total spaces and mental health symptoms when split by gender and task.

**PHQ-A Case PHQ-A Non-Case**

**Prose Composition Prose Composition**

**CAS-8 Case CAS-8 Non-Case**

dd

**Prose Composition Prose Composition**

**DQ-5 Case DQ-5 Non-Case**

**Prose Composition Prose Composition**

**ISI Case ISI Non-Case**

**Prose Composition Prose Composition**

**Table S1.**

All keystroke features.

| Dwell (Mean) | Dwell (Min) | Dwell (Skew) | Dwell (Q1) | Dwell (Median) | Spaces (Proportion) |
| --- | --- | --- | --- | --- | --- |
| Latency (Mean)* | Latency (Min) | Latency (Skew)* | Latency (Q1) | Latency (Median)* | Backspaces (Proportion) |
| Interval (Mean)* | Interval (Min) | Interval (Skew)* | Interval (Q1) | Interval (Median) | Total Non-Alphanumerics (Proportion) |
| Down Down (Mean)* | Down Down (Min) | Down Down (Skew)* | Down Down (Q1) | Down Down (Median)* | Total Null Keys (Proportion) |
| Up Up (Mean)* | Up Up (Min) | Up Up (Skew)* | Up Up (Q1) | Up Up (Median)* |  |
| Dwell (SD) | Dwell (Max) | Dwell (Kurtosis) | Dwell (Q3) | Total Keystrokes |  |
| Latency (SD)* | Latency (Max)* | Latency (Kurtosis) | Latency (Q3)* | Total Spaces |  |
| Interval (SD) | Interval (Max)* | Interval (Kurtosis)* | Interval (Q3) | Total Backspaces |  |
| Down Down (SD) | Down Down (Max) | Down Down (Kurtosis)* | Down Down (Q3)* | Total Non-Alphanumerics |  |
| Up Up (SD)* | Up Up (Max) | Up Up (Kurtosis) | Up Up (Q3)* | Total Null Keys |  |

Note: *Indicates features were removed from machine learning models before development due to high collinarity (i.e., *r* > .95).
